# Frontloading visual field tests detect earlier mean deviation progression when applied to real-world-derived early-stage glaucoma data

**DOI:** 10.1111/opo.13270

**Published:** 2024-01-16

**Authors:** Henrietta Wang, Michael Kalloniatis, Jeremy C.K. Tan, Jack Phu

**Affiliations:** 1 School of Optometry and Vision Science, University of New South Wales, Kensington, New South Wales Australia; 2 Centre for Eye Health, University of New South Wales, Kensington, New South Wales Australia; 3 School of Medicine (Optometry), Deakin University, Waurn Ponds, Victoria Australia; 4 Faculty of Medicine and Health, University of New South Wales, Kensington, New South Wales Australia; 5 Department of Ophthalmology, Prince of Wales Hospital, Randwick, New South Wales Australia; 6 Faculty of Medicine and Health, University of Sydney, Camperdown, New South Wales Australia; 7 Concord Clinical School, Concord Repatriation General Hospital, Concord, New South Wales Australia

**Keywords:** Humphrey Field Analyzer, longitudinal, perimetry, SITA-Faster, glaucoma, visual field

## Abstract

**Purpose:**

To examine the diagnostic accuracy of performing two (frontloaded) versus one (clinical standard) visual field (VF) test per visit for detecting the progression of early glaucoma in data derived from clinical populations.

**Methods:**

A computer simulation model was used to follow the VFs of 10,000 glaucoma patients (derived from two cohorts: Heijl et al., Swedish cohort; and Chauhan et al., Canadian Glaucoma Study [CGS]) over a 10-year period to identify patients whose mean deviation (MD) progression was detected. Core data (baseline MD and progression rates) were extracted from two studies in clinical cohorts of glaucoma, which were modulated using SITA-Faster variability characteristics from previous work. Additional variables included follow-up intervals (six-monthly or yearly) and rates of perimetric data loss for any reason (0%, 15% and 30%). The main outcome measures were the proportions of progressors detected.

**Results:**

When the Swedish cohort was reviewed six-monthly, the frontloaded strategy detected more progressors compared to the non-frontloaded method up to years 8, 9 and 10 of follow-up for 0%, 15% and 30% data loss conditions. The time required to detect 50% of cases was 1.0–1.5 years less for frontloading compared to non-frontloading. At 4 years, frontloading increased detection by 26.7%, 28.7% and 32.4% for 0%, 15% and 30% data loss conditions, respectively. Where both techniques detected progression, frontloading detected progressors earlier compared to the non-frontloaded strategy (78.5%–81.5% and by 1.0–1.3 years when reviewed six-monthly; 81%–82.9% and by 1.2–2.1 years when reviewed yearly). Accordingly, these patients had less severe MD scores (six-monthly review: 0.63–1.67 dB ‘saved’; yearly review: 1.10–2.87 dB). The differences increased with higher rates of data loss. Similar tendencies were noted when applied to the CGS cohort.

**Conclusions:**

Frontloaded VFs applied to clinical distributions of MD and progression led to earlier detection of early glaucoma progression.

## Key points

- Using a simulation model, frontloaded (clustered) Swedish interactive thresholding algorithm-Faster visual fields detect more cases of early glaucoma progression compared to the clinical standard of one visual field test per visit (non-frontloaded).
- Using computer simulations, where both frontloaded and non-frontloaded strategies detected glaucoma progression, the frontloaded strategy detected progression 1.0–1.3 years earlier when reviewed six-monthly and 1.2–2.1 years earlier when reviewed annually.
- Frontloaded visual field tests have a less severe mean deviation at the point of progression detection compared to the non-frontloaded strategy (0.63–1.67 dB when reviewed six-monthly; 1.10–2.87 dB when reviewed yearly).

## INTRODUCTION

Measuring glaucoma progression using visual field (VF) tests is a cornerstone of clinical care, as management decisions in glaucoma are, in large part, guided by disease trajectory.^1–3^ Currently, standard automated perimetry remains a key method for staging and prognosticating glaucoma,^4,5^ due to its close relationship with an individual's visual function.^6^

Previous studies have highlighted issues related to standard automated perimetry for progression detection, namely that the volume of data required to obtain sufficient statistical power for detection^7^ is often not met in clinical practice.^8^ Problems pertaining to statistical power for glaucoma progression detection are especially accentuated in the context of tests with greater variability and instances of data loss (where data are excluded, including due to low test reliability [noting that what is ‘reliable’ is often debated], artefacts, technical errors or other external factors), such as in fast test algorithms like the Swedish interactive thresholding algorithm (SITA)-Faster.^9^

More recently, we have shown that a frontloading strategy—performing two VF tests per eye per clinical visit—is viable for addressing issues such as data loss and provides an additional data point to potentially overcome variability issues.^10,11^ Our previous computer simulation model has shown that frontloading conducted at six-monthly visits has a much higher probability of detecting mean deviation (MD) progression in comparison to the typical clinical strategy of just one test per visit.^12^

While our previous work and that of others has stratified glaucoma into different severities of progression rate (such as slow, fast and catastrophic),^12–15^ the proportions of progressors differ by population and clinical context. Understanding how the frontloading strategy may be applied to a distribution of real-world patients would have greater clinical relevance than focussing solely on a subset of rapidly progressing patients, which are relatively rare among all glaucoma patients.^16,17^ Additionally, it is important to recognise that many patients (treated or untreated) with glaucoma do not progress or do not progress quickly (faster than −0.5 dB/year) in the short- to medium-term.^16–18^ Therefore, proposals to change a VF protocol need to account for the different distributions of progressors and non-progressors, that is, true positives, true negatives, false positives and false negatives, which until now remain poorly understood. Critically, it is important to understand how the underlying characteristics of glaucoma trajectories may impact the efficacy of the proposed frontloading strategy compared to the current clinical standard. A metric for understanding how different progression measurement techniques impact a patient is the relative difference between the initial MD (at the beginning of follow-up) and the MD at the time where progression was detected.

In the present study, we conducted a computer simulation study of MD progression when applied to two published distributions of early glaucoma patients with contrasting progression rates. We compared the clinical standard of one VF test per visit and the frontloaded strategy (two tests per visit), with the primary outcome diagnostic concordance with the pre-defined ground truth progression rate over time and thus the resultant MD at the point of progression detection.

## METHODS

This work was approved by the Human Research Ethics Committee at the University of New South Wales and adhered to the principles of the Declaration of Helsinki. Subjects in the present study had given written informed consent for their de-identified clinical data to be used for research purposes. These subjects were part of an ongoing study, the Frontloading Fields Study,^10,12^ conducted at the Centre for Eye Health, University of New South Wales and contributed to the development of the core variability model used for the computer simulation. The other component of the computer simulation study was data extracted from previously published papers (see below).

### Computer simulation model: description

**FIGURE 1:**
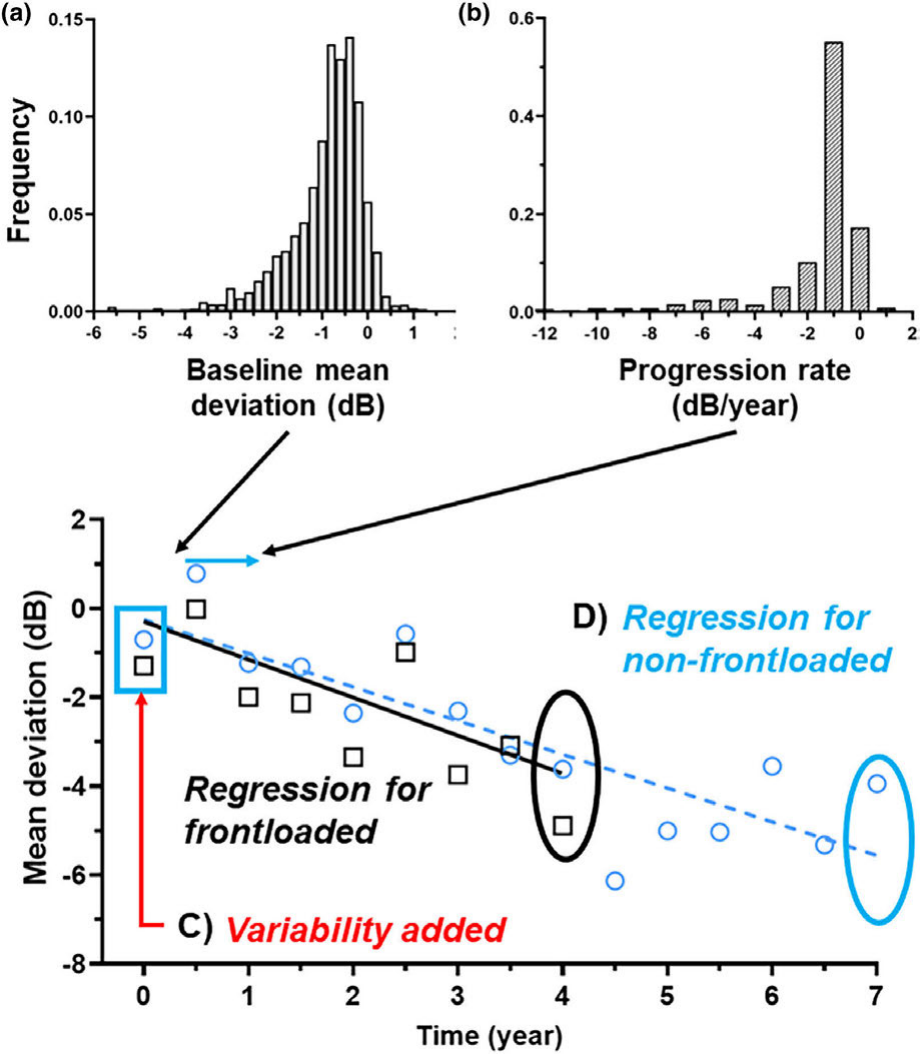
An overview of the simulation model used in the present study. Baseline mean deviation (a) and progression rate (b) were generated from a core data set. For each patient, variability was added (c; either intra- or inter-visit variability; see Methods). The simulation was conducted until the end of follow-up, and regression analysis (d) was performed at each ‘visit’ until a statistically significant negative slope was detected (or until the end of the follow-up period). Regression analysis was performed separately for non-frontloaded (blue circles only; blue dashed line) and frontloaded (blue circles plus the ‘extra’ frontloaded black squares, which were considered two separate data points; black solid line). For clarity, this figure shows a representative simulated patient in the present study with a follow-up duration of 7 years and with data points until progression was detected (year 4 for frontloaded and year 7 for non-frontloaded, as indicated by the corresponding coloured ovals).

We simulated a series of 10,000 patients followed over 10 years. This follow-up duration was chosen to ensure sufficient length to enable us to capture a potential convergence in detection rates over time that would be missed with a shorter follow-up. This was similar to our previously published model.^12^ A summary of the computer simulation model is shown in Figure 1.

A description of each simulated patient's trajectory follows. A patient first had a ground truth MD (Figure 1a) and ground truth progression rate (Figure 1b) generated from one of two previously published distributions (see below). We then generated baseline MD results, which also incorporated test–retest variability (Figure 1c). The test–retest variability (as per our previous work) was drawn from a sigmoidal function that related MD with variability specifically for SITA-Faster, whereby ‘better’ (less negative) MD values had lower variability, which increased to a plateau with increasing MD loss (see figure S1 in Phu and Kalloniatis^12^). These were then followed up at subsequent visits, each with two generated MD results until the end of follow-up. Note that each subsequent visit had two MD results, but these were not averaged. They were considered as two separate data points that were regressed, per a current clinical approach using the Humphrey Field Analyzer program. Progression analysis was performed at each visit (Figure 1d), which involved a linear regression analysis (as per a current commonly performed method in clinical practice).^19^ If the regression slope was both negative and significant at the *p* < 0.05 level, then this was deemed as progressive at that visit, and the visit number (and follow-up time) and MD at progression detection were recorded. If no MD progression was noted through the follow-up period, this was recorded as ‘non-progressive’. MD was used as the index of progression in the present study as it is a commonly used metric for staging glaucoma in clinical and research settings.^20^

### Computer simulation model: core MD and progression rates

We used two contrasting core data sets to define our patients' MD and progression rate: a published distribution from Heijl et al. (based in Sweden)^17^ and from the work of Chauhan et al. (the Canadian Glaucoma Study).^16^ The reasons for selecting two contrasting distributions are as follows: Both studies followed patients under routine glaucoma clinical care, which is useful for reflecting standard clinical practice and thus increasing clinical relevance. In comparison, clinical trials with more rigorous follow-up schedules and pre-defined treatment targets (such as the Ocular Hypertension Treatment Study^21^ or the Early Manifest Glaucoma Trial^18^), while effective for correlating variables and outcomes, may be less clinically applicable due to their relative rigour (such as in their definition of endpoints and treatment protocols). Both studies also required patients to have at least five VF test results, which enabled sufficient data for progression analysis using commercial linear regression techniques. Recognising that non-linear regression techniques have been proposed in the literature (such as by Zhu et al.^22^; also see Hu et al.^23^ for a comprehensive review of functional progression analysis in glaucoma), the validation process of the proposed frontloading model reflects clinically available linear regression methods, which are computationally simple and widely used in practice. Simultaneously, the studies have contrasting demographics and patient diagnoses. The study by Heijl et al.^17^ had a notably higher prevalence of pseudoexfoliative glaucoma, which has a more rapid progression course compared to primary open-angle glaucoma. When only using participants with a high preponderance of faster or more numerous progressors, one might expect a higher positive predictive value than would be found in routine clinical practice. Chauhan et al.^16^ also included glaucoma-suspect patients and thus represented a potentially earlier time point of the glaucoma spectrum. In analysing a cohort with fewer rapid progressors, one could offer a balanced view of the proposed VF model. Therefore, the difference in prior cohorts was important for the present study to reflect the diversity of potential clinical presentations seen in different clinical settings.

We extracted two key results from each paper: the distribution of MD and the progression rate (Figures 1 and 2 from Heijl et al.,^17^ and Figures 1a and 4 from Chauhan et al.,^16^ respectively). Since the work of Chauhan et al.^16^ included a diverse range of glaucoma severity, we limited data extraction from this study to the upper tertile of MD and progression rates (*n* = 766) to capture the primarily early stages of the disease, as the MD and progression rates are likely associated. In the present investigation, we independently sampled the baseline MD and progression rate from the given data in the two studies. Although MD and progression rates are likely related (i.e., not independent), we used independent sampling as this was the only freely accessible data from the previous papers in their published form. Data extraction was performed using DataThief (DataThief III, 2006, datathief.org).

**FIGURE 2:**
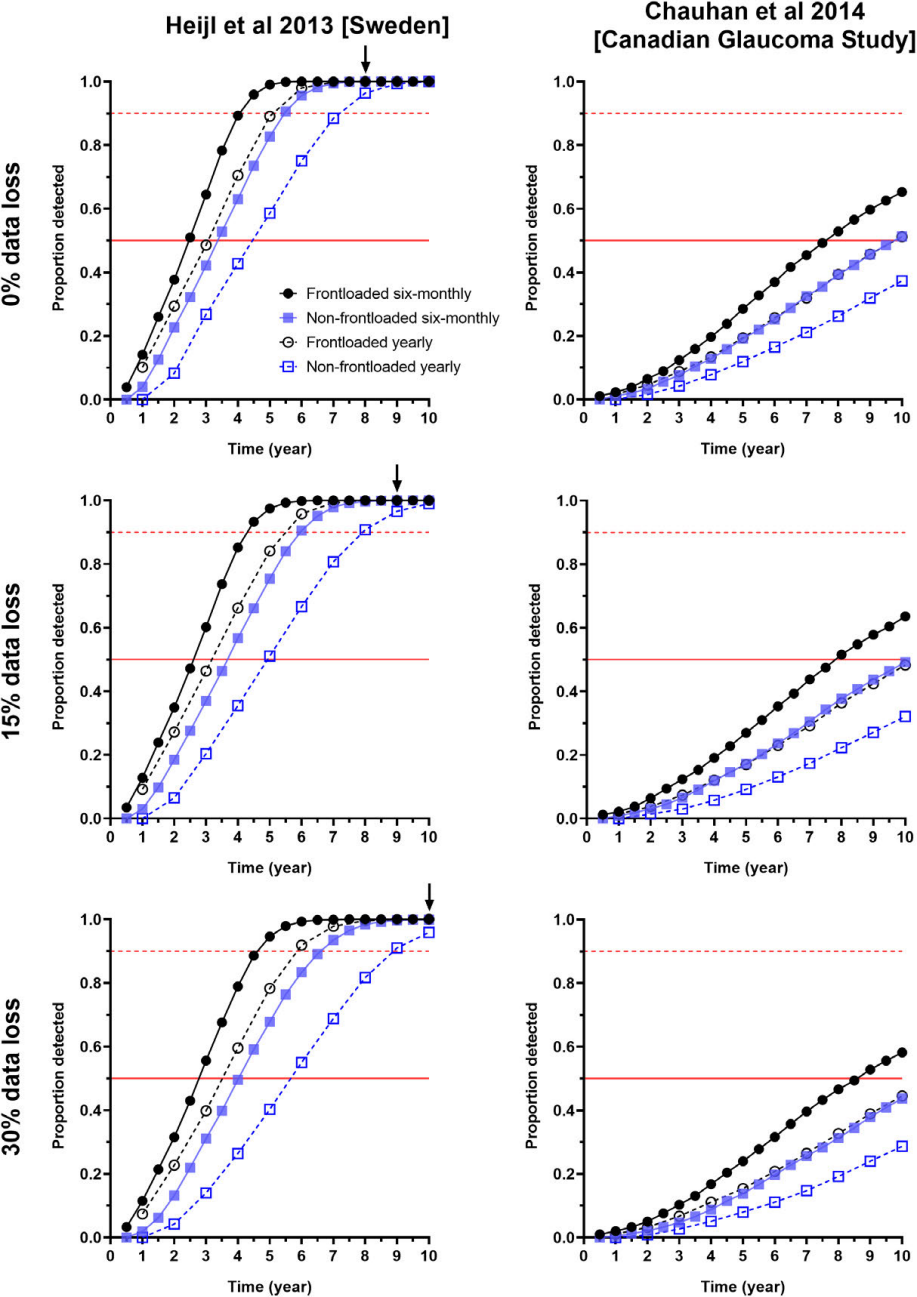
Proportion of progressors detected (*y*-axis) as a function of time (years, *x*-axis) for the two study cohorts (Heijl et al.,^17^ cohort from Sweden, left; Chauhan et al.,^16^ Canadian Glaucoma Study, right). The rows represent the different conditions of data loss (0%, 15% and 30%). The symbols represent the conditions of frontloaded (black) or non-frontloaded (blue), and the review intervals are six-monthly (filled symbols, solid lines) or yearly (open symbols, dashed lines). The solid vertical black arrows indicate the time point at which detection rates were no longer statistically significant between frontloaded and non-frontloaded strategies for the six-monthly review interval. The solid red line indicates when 50% of progressors were detected, and the dashed red line indicates when 90% of progressors were detected.

### Computer simulation model: variability

Visual field measurements are confounded by intrinsic and extrinsic variability factors,^24^ including the test algorithm. Relevant to this study, Heijl et al.^25^ demonstrated differences in retest variability among the SITA family of algorithms. In the present investigations, we used variability distributions from our recent work, which included both inter- and intra-visit estimates of variability for MD (see Tan et al.^26^ for further details on the subjects). The distributions from our work included means and standard deviations, and thus repeated samples of variability could be obtained. Additionally, the estimates of variability changed with different levels of MD (i.e., the severity of the VF defect), as previous studies have shown that perimetric variability increases (and clinical utility decreases) with greater amounts of vision loss.^27,28^ The reduction in clinical utility can be appreciated by variability-masking progression analysis. Further details for this model have been previously published.^12^

We applied variability in the present model to all simulated MD results. The first simulated VF test generated at each follow-up visit was modulated using inter-visit variability, and the second result was modulated using intra-visit variability generated from their respective distributions.

### Primary outcome and experimental conditions

Our primary outcome measure was the difference in detection rates between the non-frontloaded (clinical standard) and the frontloaded strategies. We computed *p*-values that compared the cumulative proportion detected at each time point. The test conditions under which these comparisons were made were follow-up interval (six-monthly or yearly) and data loss (low test reliability rates; 0%, 15% or 30%). Specifically, data loss represents a situation in clinical practice where VF data may need to be excluded for various reasons, such as low test reliability, artefacts, technical errors or other reasons. In the context of SITA-Faster, there may be various reasons and approaches for excluding data from progression analysis.^29,30^ In situations where data loss occurred, this was represented by the exclusion of that specific simulated VF result. Since results were not averaged, it was possible that at a given time point, there could have been two, one or zero valid VF test results for performing regression analysis, representing no data loss, one result excluded and two results excluded.

## RESULTS

We generated 10,000 simulated patients for each group of conditions and first assessed the distribution of MD and progression rate. The distribution characteristics for each cohort are shown in Table 1. Importantly, Table 1 shows that there was a proportion of subjects who, as defined by a negative MD slope, did not progress (18.4% of the Heijl et al.^17^ study and 52.6% of the Canadian Glaucoma Study^16^). Thus, this group served as the cohort for examining ‘true negatives’ and ‘false positives’ in the subsequent analyses.

**TABLE 1:** Baseline mean deviation and progression rate characteristics (median, interquartile range [IQR] and full range [FR]) used in the present simulation, obtained from two previous publications.

	Heijl et al.^17^ [Sweden cohort]	Chauhan et al.^16^ [Canadian Glaucoma Study]
Baseline mean deviation (dB)	−0.8 (IQR, −1.2 to −0.4; FR, −5.6 to +1.2)	0.0 (IQR, −0.2 to +0.1; FR, −3.1 to +1.2)
Progression rate (dB/year)	−1.0 (IQR, −2 to −1; FR, −12 to +1)	−0.01 (IQR, −1.2 to +1.2; FR, −6.1 to +6.4)

### Progression detection rates

The cumulative proportion of progressive cases detected was plotted against time (Figure 2). Across all conditions, detection rates were highest using the frontloading strategy every 6 months. Given the same review interval (either six-monthly or yearly), the frontloading strategy detected more progressors at an earlier time point. When considering the cohort from Sweden (Figure 2, left column), there was a plateau of detection, whereby all progressors were eventually detected by all methods. However, with increasing rates of data loss, the non-frontloaded strategy had reduced detection rates, while the frontloaded strategy appeared less affected. Using the six-monthly follow-up interval, at the year 8, 9 and 10 time points for 0%, 15% and 30% data loss conditions, there was no longer a significant difference in detection rate between frontloaded and non-frontloaded methods (*p* > 0.05). For all other combinations of conditions, the frontloaded strategy detected more progressors at all time points (*p* < 0.01 for all).

A similar result was found when examining the Canadian Glaucoma Study cohort (Figure 2, right column). However, in this cohort, the detection rate for the non-frontloaded strategy performed six-monthly was essentially equivalent to frontloading yearly. Furthermore, the overall detection rates were lower, with no obvious plateau and did not reach 100% of the progressors within the simulated 10 year period. Accordingly, the frontloaded strategy detected significantly more progressors at all time points (*p* < 0.0001).

### Relative detection rates

**FIGURE 3:**
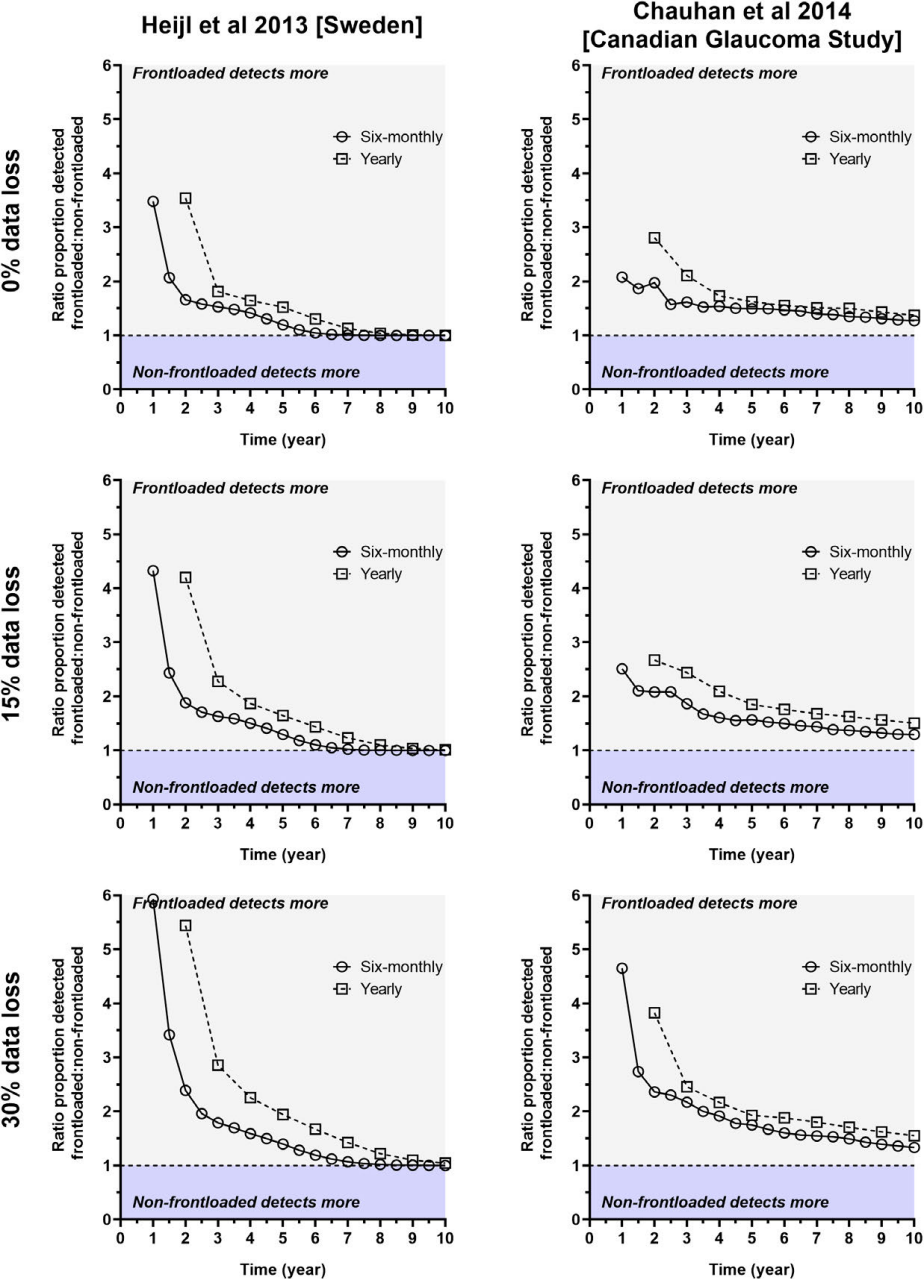
Ratio of the proportion of progressors detected when using frontloading compared to the non-frontloaded strategy (*y*-axis) as a function of time (years, *x*-axis). The columns indicate the core data and the rows the data loss conditions, as per Figure 2. As described in the text, a ratio >1 indicates that the frontloaded approach detected more progressors (grey shaded zone), a ratio of 1 indicates parity between methods (black horizontal dashed line) and a ratio <1 indicates that the non-frontloaded approach detected more progressors (light blue zone). The open circles and black solid line indicate the results for six-monthly follow-up, and the open squares and dashed line indicate the results for yearly review.

We took the data from Figure 2 and calculated the ratio of proportion detected when comparing the frontloaded and non-frontloaded strategies. A ratio >1 favours the frontloaded strategy, 1 indicates parity and <1 favours the non-frontloaded strategy. Figure 3 shows these results and demonstrates that the frontloaded method was markedly favoured in the first few years of follow-up, asymptoting towards parity in later follow-up visits. Notably, since the detection rate did not reach 100% for the cohort from Chauhan et al.,^16^ the asymptote did not reach a ratio of 1. As expected from Figure 2, the relative ratio was higher under conditions of data loss.

**FIGURE 4:**
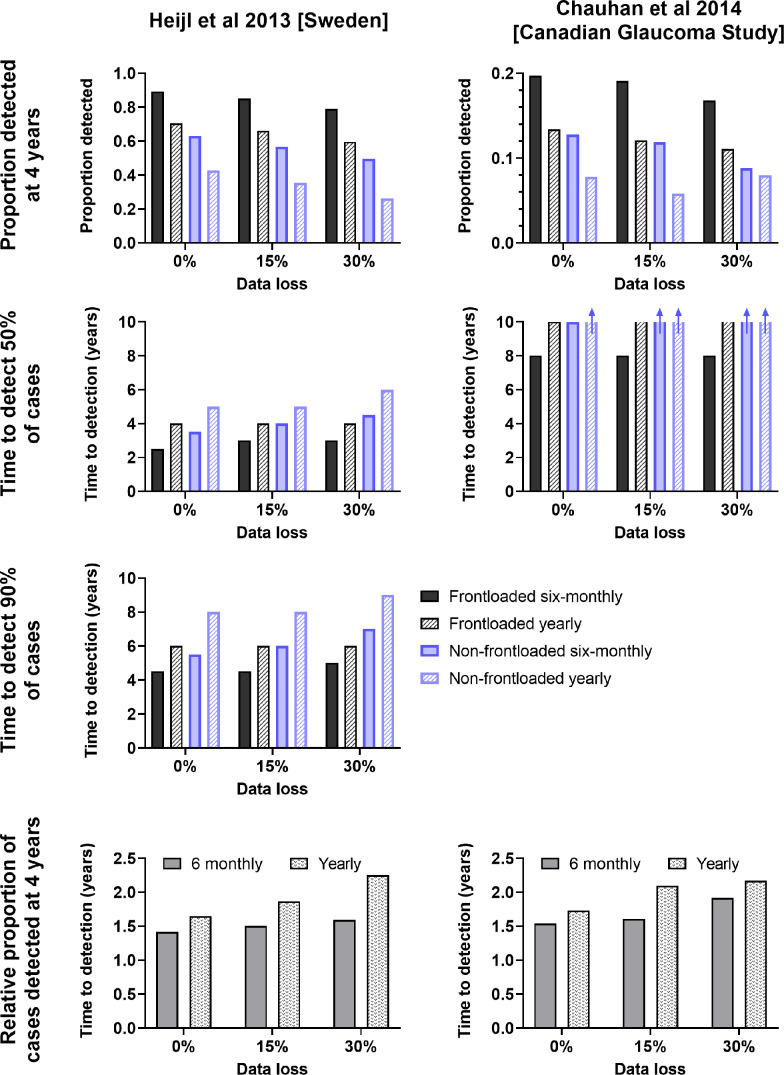
Summary of the key data and outcomes extracted from Figures 2 and 3.

Three parameters of interest (proportion of progressors detected at 4 years, year at which at least 50% of progressors were detected and year at which at least 90% of progressors were detected) are shown in Figure 4, serving as a summary of key clinical parameters of interest.

### Distribution of outcomes across the follow-up period

**FIGURE 5:**
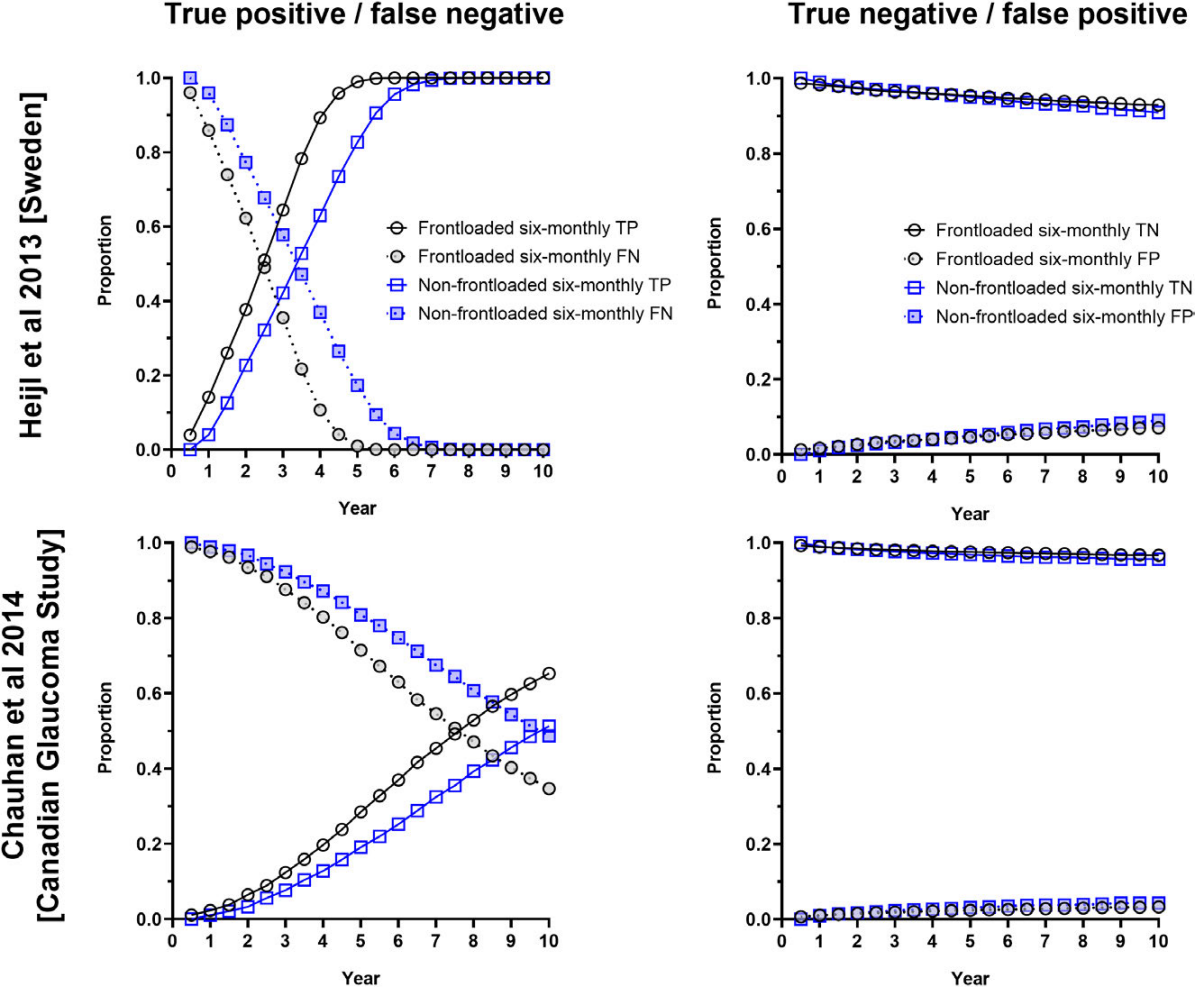
Proportions of true positive and false negative (left) and true negative and false positives (right) for the two study cohorts (Heijl et al.^17^ from Sweden, top and the Canadian Glaucoma Study,^16^ bottom). The black circles indicate the frontloaded method, and the blue squares indicate the non-frontloaded method. The open symbols and solid lines indicate the true positive (left) and true negative (right) rates, and the filled symbols and dashed lines indicate the false negative (left) and false positive (right) rates.

In addition to examining the cumulative detection rate, we also examined the true positive, false positive, true negative and false negative rates across the duration of follow-up. The results for the six-monthly reviews and 0% data loss condition are shown in Figure 5. At all time points until the plateau, the frontloading strategy had a higher true positive and lower false negative rate compared to the non-frontloaded method. There was no discernible difference in cumulative true negative and false positive rates, which, as expected, tended to increase over time. As shown in Figure 3, true positive rates did not reach a notable plateau for the Canadian cohort, most likely due to the reduced ability to detect progression in relatively slower progressors.

### Difference in time point at which progression was detected

**FIGURE 6:**
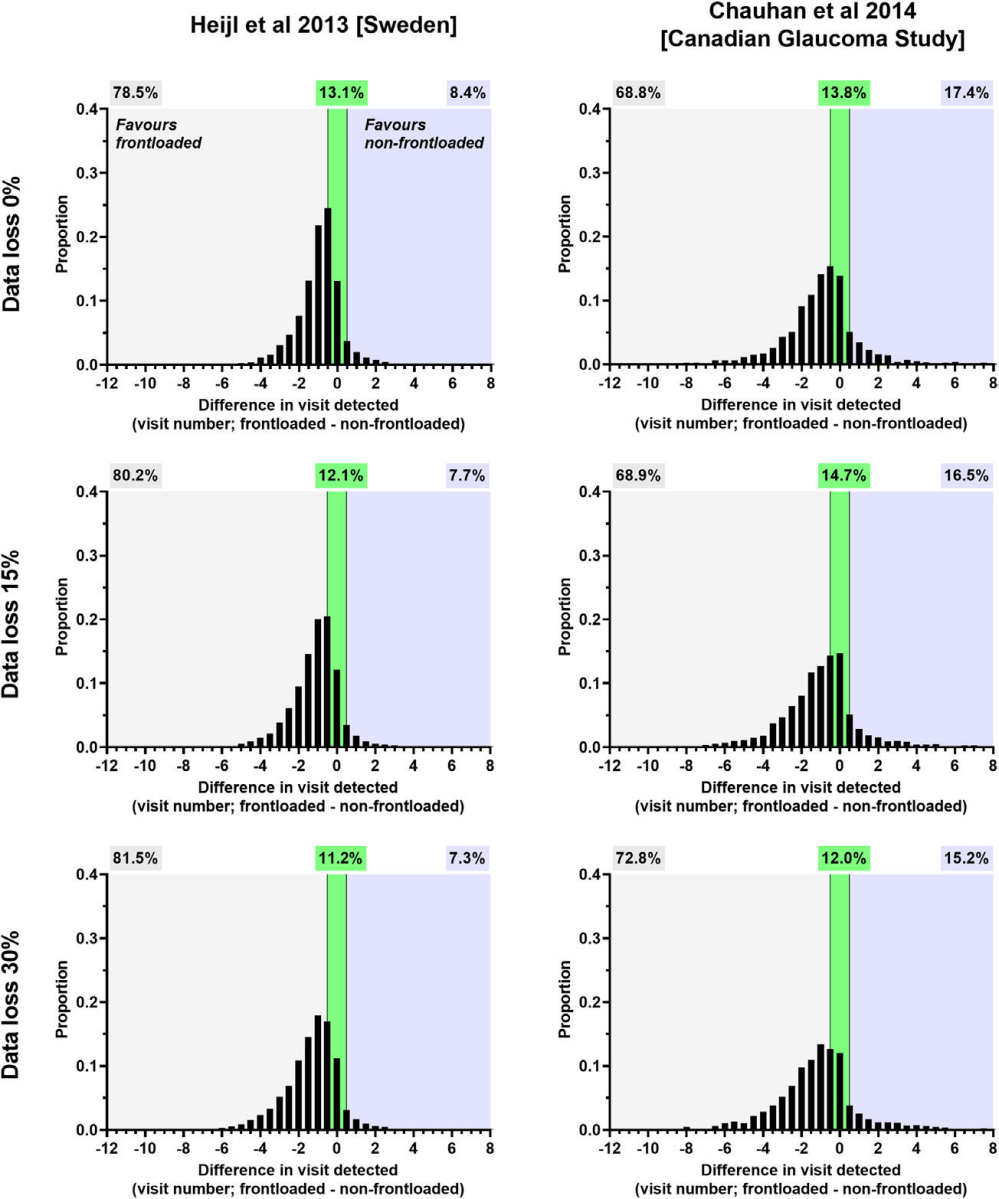
Distribution of the difference in visits where mean deviation progression was detected (frontloaded—non-frontloaded) for each test condition for six-monthly reviews. A negative *x*-axis value means that the frontloaded strategy detected progression at an earlier visit compared to the non-frontloaded strategy (light grey zone), and a positive *x*-axis value represents the opposite (light blue zone). The relative proportions are indicated by the correspondingly shaded cells above. The green zone represents no difference in visit of detection between methods.

**FIGURE 7:**
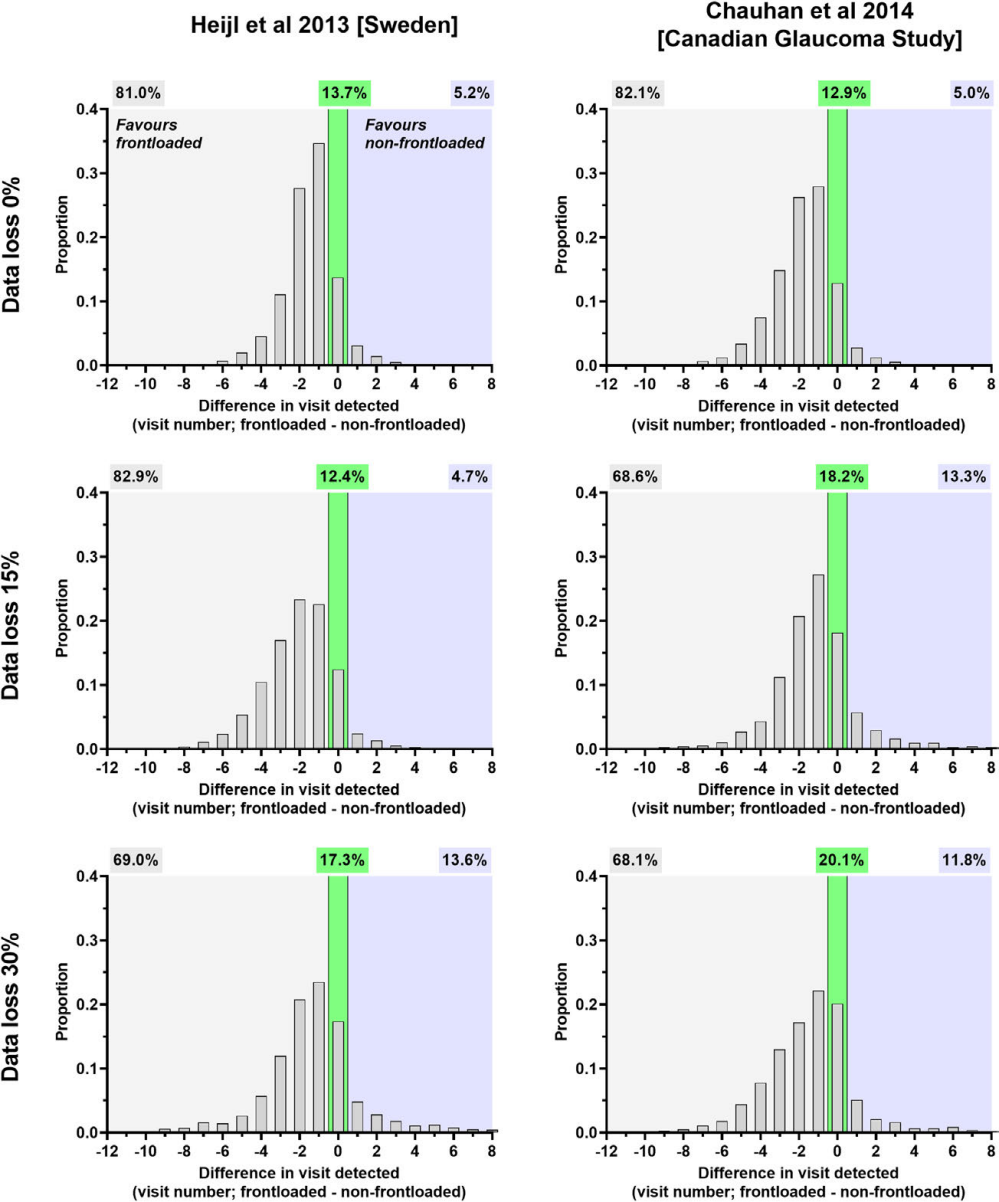
Distribution of the difference in visits where mean deviation progression was detected (frontloaded—non-frontloaded) for each test condition for yearly reviews. A negative *x*-axis value means that the frontloaded strategy detected progression at an earlier visit compared to the non-frontloaded strategy (light grey zone), and a positive *x*-axis value represents the opposite (light blue zone). The relative proportions are indicated by the correspondingly shaded cells above. The green zone represents no difference in visit of detection between methods.

For simulated patients who had MD progression detected by both frontloaded and non-frontloaded strategies, we determined the difference in the time point at which detection was noted as measured by the visit number. The results for the six-monthly and yearly follow-up protocols are shown in Figures 6 and 7, respectively. Several findings were evident. Under all conditions, on average, the frontloaded strategy detected the progressors sooner than the non-frontloaded strategy, with a difference of 1.0–1.3 years when reviewed six-monthly and 1.2–2.1 years when reviewed annually (one-sample *t*-test, *p* < 0.0001 for all). For the Heijl et al. cohort, 78.5%–81.5% (six-monthly follow-up) and 81%–82.9% (yearly follow-up) of patients had their MD progression detected first by the frontloaded strategy. The higher proportions were seen under conditions of greater data loss. A similar finding was found for the Chauhan et al. cohort.

### Difference in MD value at the point of progression detection

**FIGURE 8:**
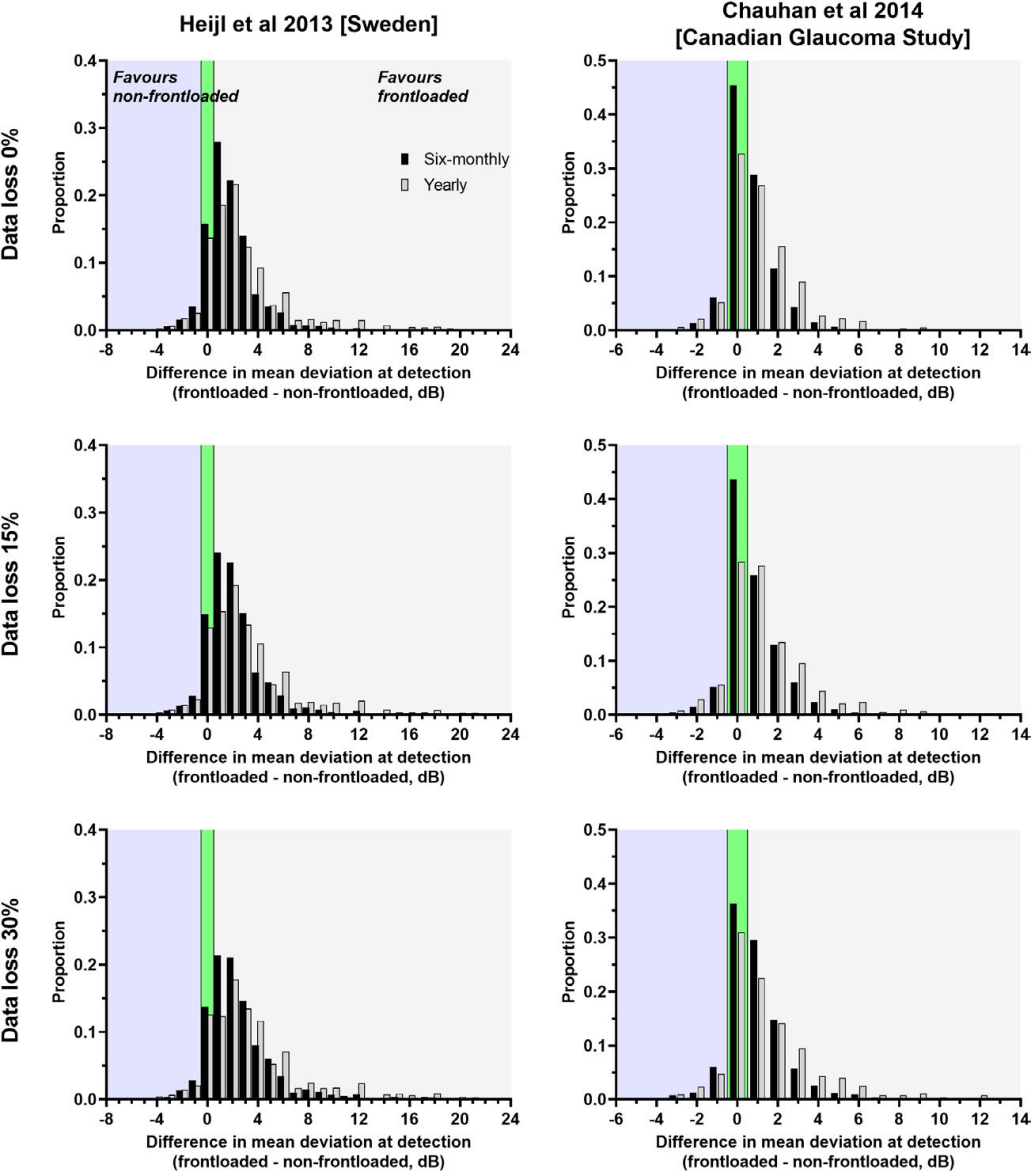
Distribution of the difference in mean deviation at detection (frontloaded—non-frontloaded) for each test condition (black bars, six-monthly follow-up; grey bars, yearly follow-up). *X*-axis bins are in integer values, with the data bars jittered slightly for clarity. A positive *x*-axis value means that the frontloaded strategy detected progression at an earlier visit compared to the non-frontloaded strategy (light grey zone), and a negative *x*-axis value represents the opposite (light blue zone). The green zone represents no difference in visit of detection between methods.

Using the data from Figures 6 and 7, we then calculated the MD value at the visit at which progression was detected by each method. This was based on the ground-truth progression rate. These results are shown in Figure 8. Similar to the findings when examining the difference in the time point of detection, patients who were detected using the frontloaded strategy had, on average, a less severe MD compared to the non-frontloaded strategy (*p* < 0.0001 for all conditions). The difference was greater with yearly follow-up and under conditions of data loss, as predicted from the difference in visit of detection. For the cohort reported by Heijl et al., under conditions with 0% data loss, the MD difference was 1.67 and 2.87 dB for six-monthly and yearly follow-ups, respectively. The differences increased to 2.36 and 3.73 dB for six-monthly and yearly follow-ups, respectively, when there was 30% data loss. A similar tendency was found for the Chauhan et al. cohort, although with slightly lower magnitudes of differences (0.63 and 1.10 dB for six-monthly and yearly follow-ups, respectively, in the context of 0% data loss).

**FIGURE 9:**
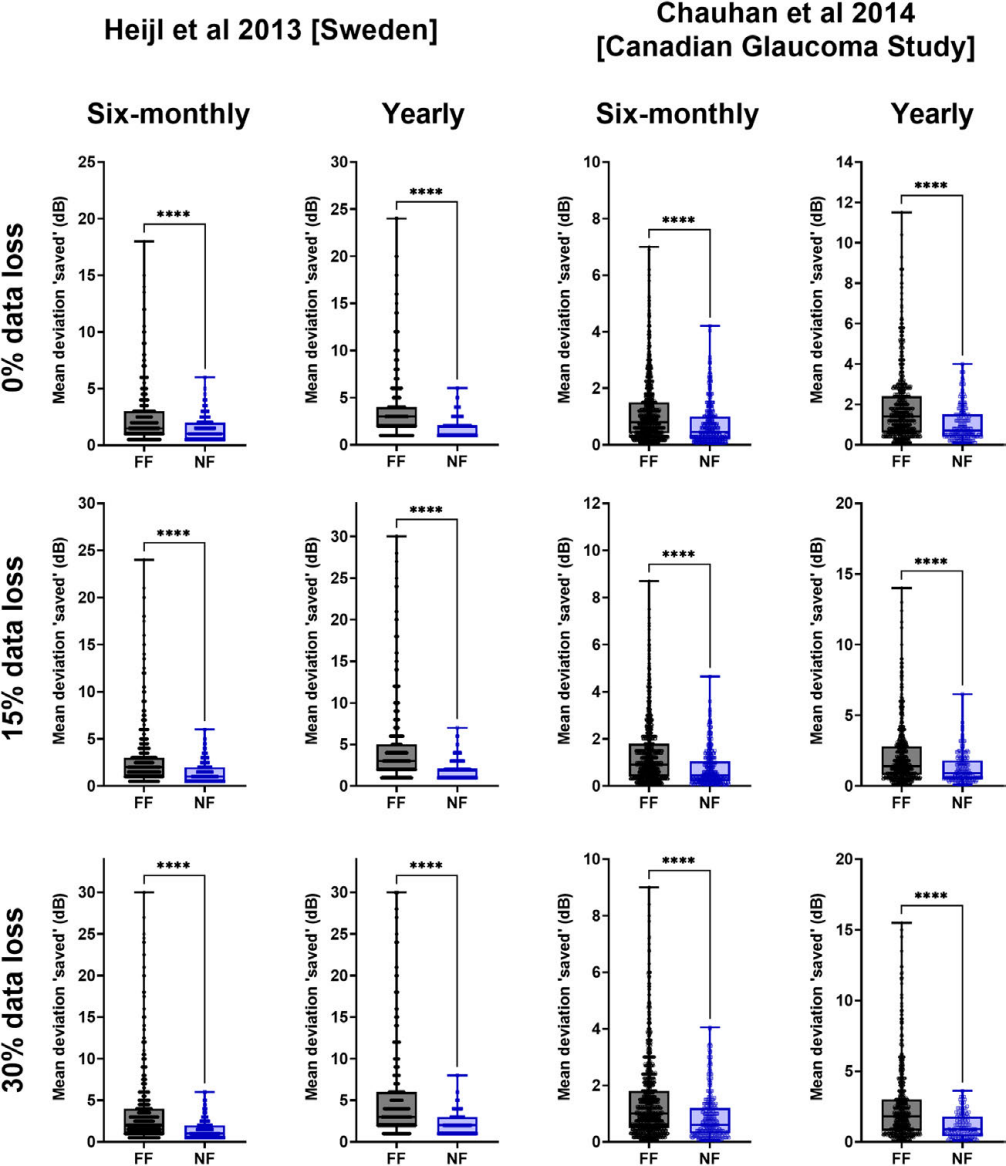
Box-and-whisker plots of the amount of mean deviation (MD) ‘saved’ (the difference in MD at detection) when favouring either frontloaded (FF) or non-frontloaded (NF) strategies as per Figure 8. A higher *y*-value indicates more MD ‘saved’ (less vision lost). The asterisks indicate significant differences in the distribution. Note that the *y*-axis limits are different due to the differences in range, which allowed for better visualisation of the data.

The MD results presented in Figure 8 and described above were for all cases detected by each strategy. However, we also determined the difference in MD at the point of detection in cases that were detected by both methods to compare their differences directly. These results are shown in Figure 9. For all conditions, the MD ‘saved’ was higher when frontloading detected progression prior to the non-frontloaded method (*p* < 0.0001 for all). Although the median difference was relatively small, as per Figure 8, there was a clear skewed distribution where many more cases of frontloaded VFs tended to result in larger amounts of MD ‘saved’, especially under situations of significant data loss.

## DISCUSSION

In the present study, we examined the effects of frontloading VFs (clustering VF testing within the same visit) in a model that incorporated data from real-world populations of glaucoma patients with long-term follow-up. In comparison to previous studies examining only subsets of progression rates, the approach of drawing from published longitudinal distributions of glaucoma incorporates pre-test probabilities of progression that are therefore more reflective of clinical populations. This study produced several key findings. First, the frontloaded strategy (clustered VF testing within the same visit) identified more progressors compared to the clinical standard of performing one VF test per visit, with the increase in progression detection observed across varying rates of progression and not just being limited to rapid progressors, with no appreciable difference in false positive rates between techniques. Second, in cases where both techniques detected progression, the frontloaded strategy detected progressors earlier compared to the non-frontloaded strategy (78.5%–81.5% and by 1.0–1.3 years when reviewed six-monthly; 81%–82.9% and by 1.2–2.1 years when reviewed yearly), and, accordingly, these patients had less severe MD scores (0.63–1.67 dB ‘saved’ when reviewed six-monthly; 1.10–2.87 dB when reviewed yearly for 0% data loss). Third, the ‘benefit’ to detection and MD ‘saved’ was greater under conditions of greater data loss. The results also demonstrated that patients with a preponderance for slower progression (the Canadian Glaucoma Study) were challenging to detect as progressing on MD even with frontloading. However, despite this limitation, frontloading six-monthly significantly improved the detection rate (approximately 50% even at 10 years) compared to the current clinical standard.

### Increasing the amount of data raises the likelihood of detecting progression in clinical distributions

The benefits of frontloading for detecting progression observed in this study were consistent with previous work showing that increasing the number of VF tests improves the statistical power to detect change.^7,12^ Specifically in slow progressors, we recently demonstrated that frontloading also affords benefits in identifying more cases of progression. However, notably with very slow progressors, there was a proportion of people who continued to be missed.^15^ In the present study, we applied similar principles of frontloading, but to a cohort that aimed to reflect the clinical distributions of MD and its progression rates in glaucoma. Early to moderate glaucoma was chosen specifically for this study, as there is interest in capturing this group of progressors to prevent progression to more advanced vision loss, while simultaneously it is acknowledged that perimetry, in its current form, is relatively less effective for progression analysis compared to other strategies such as optic disc examination and optical coherence tomography, especially in early glaucoma.^31,32^

Although both cohorts showed the benefits of the frontloaded strategy, their results differed in the final proportion of progressors detected. Heijl et al.^17^ reported on a cohort that had a higher prevalence of pseudoexfoliative glaucoma and were generally faster progressors. Unsurprisingly, this meant a greater likelihood to detect progression with a faster rate of change, and in the case of the clinical standard paradigm, eventually catching up to the frontloaded method. Conversely, the cohort reported by Chauhan et al.^16^ generally exhibited slower progression, and accordingly, there was no plateau and detection rates did not reach 100% within the 10-year time frame. This was also consistent with one of our recent studies, where VF progression in slowly progressive glaucoma was particularly challenging to detect, which used a cohort of patients with predominantly early glaucoma for developing the model.^15^ Lower detection rates have been attributed to variability exceeding the progression rate, thus masking a significant slope change.

The clinical interpretation of the relative difference in detection rate therefore depends on the likelihood of rapid or slow progression and needs to be interpreted contextually, given differences in distributions of progression rate.^33^ In situations where there is a high pre-test probability of rapid progression, such as pseudoexfoliative phenotypes,^18^ frontloading is expected to provide an earlier diagnosis, with non-frontloading catching up approximately two visits later. In situations where the pre-test probability suggests slow progression, such as pre-perimetric normal-tension glaucoma, frontloading provides a higher probability for progression detection, even at later time points.

### False positive rates are similar between frontloaded and non-frontloaded strategies

In the present study, we found only a small but not significant elevation in false positive rate when performing one compared to two VF tests per visit, which is similar to previous reports of frontloading or clustering perimetric data.^12,34^ Obtaining additional perimetric data in this situation was hypothesised to improve the robustness of the average MD at each visit, thereby reducing the false positive rate. Similarly, when focussing on the context of the clinical cohorts, pre-test probabilities that suggest a low risk of rapid progression (e.g., in the Canadian Glaucoma Study) mean that the likelihood of a false positive is initially low for both strategies. Thus, it remains reassuring that the frontloaded strategy did not differ significantly from the current clinical standard in terms of false positive rate.

### Time and MD ‘saved’

In addition to detecting *more* cases of progressors, the data then revealed that in patients in whom detection was found using both methods, the frontloading strategy detected progression sooner than the not-frontloading methodology. This earlier detection implies less vision loss, the magnitude of which depends on the underlying progression rate. On average, this was a relatively small difference in MD. However, for some patients, this meant a transition between different stages of glaucoma. The distributions also highlighted greater differences with increasing data loss. This emphasises the importance of data integrity and continuity in facilitating the ability to detect progression.

This study focussed on MD as the index of glaucoma progression and beneficial outcome when comparing methods, but this can be further examined using other metrics such as quality-of-life, visual functioning^35^ and financial cost,^36,37^ which have also been linked to different progression strata. While we have previously shown that the absolute cost in time is minimal at the level of the individual patient, over time and for many patients, this cost is expected to accumulate. This can be assessed in a future study.

### Limitations

The limitations of a model for progression detection have been discussed previously, as we used a model similar to our previous study.^12^ One of the fundamental assumptions related to glaucoma progression is the linearity of MD change over time, which is how it is expressed in clinician-facing devices. Hu et al.^23^ have provided a comprehensive review of functional progression analysis in glaucoma, including an overview of event-based and trend-based analyses and subtle variations within each method that may be complementary for progression detection. Importantly, many such approaches are not clinician-facing at present. Specifically, we highlighted that alternatives such as sigmoidal functions have been proposed to pointwise data to characterise different rates of change throughout the range of glaucoma severity.^38^ Over short-term follow-up, this assumption may be reasonable, as many clinical trials, such as the studies from which our core data were extracted,^16,17^ reported outcomes over a relatively short period of time. This model also does not incorporate other factors that may contribute to the pre-test probability of glaucoma progression (such as secondary phenotypes and intraocular pressure), as the goal was to identify functional change over time. Although we used core MD and progression data from studies reporting on patients under clinical care, we acknowledge the diversity of presentations and management plans and therefore the disease trajectory across different clinical settings.

The present study used a computer simulation model, and although we used core data sourced from clinics and external clinical data, the outcomes still require evaluation using empirical real-world longitudinal data collected in patients to confirm the external validity. The design of this computer simulation meant that we could retrospectively extract the visit at which progression was identified using each technique and the discordance between early and later detection methods. This method would not be conducive to clinical practice, as progression would typically trigger treatment change. Thus, an empirical study would require a different study design.

We limited our study to SITA-Faster, which has been proposed as an alternative to SITA-Standard. SITA-Fast parameters were not used as previous methods for frontloading or clustering VF data in real-world clinical settings.^10,39^ SITA-Fast has different variability characteristics compared to SITA-Faster, which would warrant a different study. Additionally, although we used a large volume of data to understand the variability characteristics of SITA-Faster with respect to its MD outputs, there remain substantial unknowns regarding its inner workings. An example is the spatial smoothing of the result, which is an important factor when considering the progression of clusters of VF defects over time. Though this may not impact MD significantly in the context of the present analysis, future studies examining pointwise change would need to understand this aspect of SITA-Faster to better understand and model progression analysis.

### Future directions

We limited our analyses to the 24-2 test grid, recognising that there are other grids available in clinical practice, such as the 24-2C and 10-2. A practical reason for this choice was the lack of longitudinal clinical study data on glaucoma progression with multiple VF tests using the 24-2C and 10-2 with the level of follow-up reported by the two core studies examined here. We have previously demonstrated comparable global indices between the 24-2 and 24-2C,^40^ and thus, a future study, given the appropriate data, could investigate the role of the additional macular test locations in the 24-2C to progression analysis using pointwise and global indices.

The present study was limited to the analysis of glaucoma, which is arguably the most common clinical indication for performing 24-2 perimetry in practice. However, similar principles of perimetric testing could be assessed in non-glaucomatous ocular and neurological pathology. This is a potential topic for further studies.

We previously described the merits of a longer, singular, interleaved test that returns more ‘accurate’ results or data useful for perimetric analysis.^10,12^ This concept, resembling that of ‘fluctuations’ in older perimetric paradigms, would require a customised test paradigm and significant further research, development and validation prior to implementation.

Finally, although we first termed this approach frontloading in the present and our earlier publications, we recognise that this may be misinterpreted as only performing multiple tests at the baseline visit. Accordingly, we have suggested alternative nomenclature—intra-visit clustering—analogous to the ‘wait and see’ approach, but this could also refer to VF tests performed at different visits closely spaced apart. Another term could be ‘dublet’ VF testing, as the implication would be that double testing could consolidate perimetric analysis. In the future, further refinement of the preferred terminology could facilitate greater clinical uptake in the community.

## CONCLUSIONS

Using real-world published data of glaucoma patients under routine clinical care, we found that frontloading (doing two VF tests per eye per visit) identified more cases of progression at an earlier time point compared to the current clinical standard of one test per visit, thus potentially allowing time to intervene to prevent further vision loss. Based on this work, a clinical recommendation is to perform at least two VF tests per visit per eye to assist in identifying glaucomatous progression using MD, especially in the context where data loss may be expected.
